# Increased Range of Motion and Function in an Individual with Breast Cancer and Necrotizing Fasciitis—Manual Therapy and Pulsed Short-Wave Diathermy Treatment

**DOI:** 10.1155/2010/179581

**Published:** 2010-07-14

**Authors:** Wayne Johnson, David O. Draper

**Affiliations:** Brigham Young University, Provo, UT 84602, USA

## Abstract

Necrotizing fasciitis is a severe soft tissue infection of the subcutaneous tissue and fascia affecting those predisposed to immune system compromise. It is a life threatening condition; mortality can be reduced by rapid diagnosis, adequate early surgical debridement and antibiotic ointment. In this case report we present the use of manual therapy (MT) techniques, joint and soft tissue mobilization, following a regimen of pulsed short wave diathermy (PSWD) in the treatment of a woman 3 years post necrotizing fasciitis developed during chemotherapy treatment for breast cancer. During her course of chemotherapy, she developed necrotizing fasciitis which was treated with extensive surgical debridement (8 linear feet of incisions) followed by debridement to both hips and the pelvis area. When we started working with her, we put her on a course of PSWD/MT. After six weeks of following this regimen, she gained 25° of external rotation in both her left and right hips, 15° of left hip flexion and 17° of right hip flexion. The patient gained 10° of right hip extension, yet there was no improvement in left hip extension. The treatments led to a dramatic reduction in pain and scarring from previous surgeries. The patient also returned to running.

## 1. Background and Significance

Physical therapy intervention for individuals after breast cancer is an important area of practice, especially as the prevalence of cancer survivors increases. Physical therapy addresses deficits in range of motion (ROM), strength, posture, and function. Manual therapy is used to minimize scar tissue, fibrosis, or adhesions. Lymphedema-prevention education and treatment for lymphedema is provided. Complications experienced during the treatment of cancer can further impact a patient's functional outcome. Infection is one such complication. Severe infection, such as necrotizing fasciitis (NF), is life threatening for the patient [1–3]. Patients who survive the infection may experience severe scaring and limited function [2]. Treatment of scaring and limited function is important to the quality of life of the individuals with these issues, but individuals may not be directed to treatments that can benefit their condition. This may be because so much effort is given to save the life of the individual that consideration for later rehabilitation is diminished. Increasing understanding of the role that rehabilitation plays in improving function and quality of life of individuals post breast cancer with surgical debridement for necrotizing fasciitis is significant to medical literature. 

Necrotizing fasciitis is a severe soft tissue infection of the subcutaneous tissue and fascia affecting all age groups predominately predisposed to immune system compromise [3]. It is a life-threatening condition typically treated with resuscitation and aggressive debridement of necrotic tissue [1–3]. Mortality form necrotizing fasciitis can be reduced by rapid diagnosis, adequate early surgical debridement and antibiotic treatment [3]. Data about the long term outcomes of surviving the infection are limited.

In this case report we present the use of manual therapy techniques for joint and soft tissue mobilization, following a regimen of pulsed shortwave diathermy (PWSD) in the treatment of a woman 3 years status post necrotizing fasciitis developed during chemotherapy treatment for breast cancer. This individual experienced a dramatic increase in ROM and self-reported function following treatment.

## 2. Patient History and Review of Systems

The subject was a 27-year-old woman (height =1.65 m, weight = 61.2 Kg, and BMI = 22.5), who was 3 years post breast cancer with complication of necrotizing fasciitis. She underwent mastectomy and chemotherapy treatment. During the course of chemotherapy she developed necrotizing fasciitis, which was treated with extensive surgical debridement (2.4 m or 8 linear feet of incisions). Surgical debridement was done bilaterally to the hips and pelvis (posterior thigh and gluteal region of the right hip and medial proximal thigh of the left hip, each extending at least 2/3 the way down the thigh), as well to portions of the upper extremity and trunk. She was hospitalized for 2 months following the surgery, and she later received additional surgical procedures, including hysterectomy. The subject experienced severe pain and scaring. Pain levels were reported to range from 5 to 8/10 without pain medication on the visual analog scale [4] during rest, increasing with activities, such as running. Due to the extensive scaring sustained during the surgical procedures, and soft-tissue contracture and adaptive shortening developed over years of altered motion and postures to avoid pain lead to decrease in functional movement. The lack of normal movement leads to muscle tightness progressing to trunk and pelvic asymmetries. Avoidance of pain further reinforced the persistence of the trunk and pelvic asymmetries by hindering functional ROM. The asymmetries in the tissues of the hip, trunk, and pelvis produced pelvic girdle and lumbar dysfunctions. These dysfunctions hindered her function and care of her children. She sought medical care from multiple medical specialties for three years without significant improvement in her condition, except for physical therapy treatment for elbow flexion/extension restriction. Treatment for the soft-tissue contracture at the elbow joint included ASTYM (Performance Dynamics, Inc.), which is a soft-tissue mobilization technique that involves running an instrument firmly along the skin, following the direction of the muscle, tendon, or ligament in order to break up scar tissue and adhesions. The subject had a return of ROM and improved function in the arm. The subject's insurance coverage for physical therapy was exhausted during these treatments and no treatment to trunk, pelvic girdle, and hip was provided. The subject did not complain of ROM restrictions of her shoulders or upper extremity at the time of the current study, and therefore ROM was not measured.

## 3. Examination

At start of treatment the subject had asymmetrical restricted trunk and hip ROM. The subject's shoulders and upper extremities did not have a restriction of motion nor did the subject report pain in those areas currently. She had altered posture of the hip, pelvis, and lumbar spine. She had leg length discrepancy (LLD) of 2.5 cm and wore a heel lift. Leg length was measured from anterior superior iliac spine to medial malleolus; it was also visually noted by comparison of bilateral malleoli with the subject in supine. Her skin over the affected sites was very tight and rigid compared to the unaffected areas. She had pain with walking and running. Active ROM was measured according to methods described by Norkin and White [5] with the use of a standard goniometer in a supine or prone position for the various ROM measurements. Each measurement was taken three times by the same evaluator at each assessment and then averaged. Intrarater reliability of goniometry measurement has been found to be good to excellent (ICC > 0.80) [6]. Hip ROM was: left external rotation: 20°, right external rotation: 5°, left flexion: 115°, right flexion: 128°, left extension: 25°, right extension: 15°. Differences in restriction of motion were noted between right and left hip measurements likely due to location of surgical debridement and variances in the formation of scar tissue and soft-tissue contractures.

## 4. Intervention

The subject was treated for 6 weeks, 3 times a week for the first 3 weeks and then 1 or 2 times per week. Treatment included PSWD (Megapulse II, Accelerated Care Plus Corp. Reno, NV) at 48 Watts, 20 minutes to each upper thigh, over the proximal femur, hip, and buttock. These parameters were used, because previous studies showed that muscle temperature increased by 4-5°C at 3–5 centimeters depth [7]. Two diathermy units, each with a 15 cm drum head were used to treat both the left and right simultaneously. This vigorous temperature increase enabled us to work on increasing her ROM and reducing her scar tissue. Diathermy was deemed to be safe for this subject, because the subject was 3 years post breast cancer, had a hysterectomy, and it was only applied to the upper thigh, hip, and buttocks at the areas indicated by the subject as painful or restrictive of motion. Manual therapy joint mobilization, including the hip (distraction and gliding motions grades 2 to 4 on the Maitland scale of joint mobilizations) and muscle energy techniques, were performed as was soft tissue mobilization prior to active and passive ROM and gait instruction. Muscle energy techniques used were similar to those advocated by Greenman [7]. Specific techniques used include treatment dysfunctions of the innominate, lumbar, and sacroiliac joints. These included upslipped innominate, rotated innominate, symphysis pubis, inflared/outflared innominate, and sarcoriliac dysfunctions. The study participant was assessed and treated for the specific dysfunctions noted in each assessment session. These assessments followed the methodology suggested by Greenman [7]. Muscle energy techniques were done by first assessing the patient's condition. Once the dysfunction was identified the patient was positioned for the specific treatment, which involved moving the legs and/or trunk to isolate motion to a specific joint and to engage the resistive barrier (the barrier that prevents movement in the direction of motions loss) [7]. The patient was asked to move out of the resistive barrier while the clinician resisted the motion resulting in an isometric contraction of the patient's muscles. The isometric contraction was held for 3 to 5 seconds and each technique is repeated 3 times each treatment session. This isometric contraction resulted in a mobilization of the joint secondary to the patient's own effort. For example, in a posterior rotated innominate the patient would be positioned supine. The affected leg would be lowered off the edge of the plinth until the resistive barrier is felt by the clinician. The patient would then be asked to produce a hip flexion effort moving out of the resistive barrier. The clinician resists the hip flexion resulting in the patient performing an isometric contraction. The contraction is held for 3 to 5 seconds, the leg is then moved again toward the resistive barrier taking up the additional motion produced in the previous effort. This procedure is repeated 3 times. The patient is then reassessed. The effort by the patient produces an anterior movement of the innominate, thus, eliminating the posterior innominate dysfunction. Each dysfunction was treated as needed during each visit. Treatment order of the dysfunctions followed the order suggested by Greenman [7]: the pubic symphysis, upslipped innominate, sacroiliac dysfunctions, followed by iliosacral dysfunctions. 

Soft tissue mobilization and passive ROM stretches were performed by the treating physical therapist. The subject was given a home exercise program, including active and passive stretching to the hips and low back. Passive ROM exercises were held for 30 seconds and repeated three times each session, each stretching session was repeated two times per day. Gait was practiced by the physical therapist observing the subject's gait pattern and providing verbal cueing to help normalize the pattern. This occurred as the subject walked 7 to 10 meters 8 to 10 times. The total treatment time ranged from 60 to 90 minutes including diathermy, manual therapy, and exercises.

## 5. Outcome

The subject had a clinically significant increase in joint ROM (see Table 1). Leg length was equal between legs and she no longer required a heel lift, this change in leg length primarily occurred during the first 3 weeks of treatment and then was maintained throughout remaining treatment duration. Pain during gait was eliminated. She reported a softening of tissues around her scar. The subject returned to running. She reported improved function at home and a decrease in night discomfort. Hip ROM increased as shown in Table 1.

## 6. Discussion

The subject in this study showed improvement in ROM and reported improvement in function with a dramatic decrease in pain. The pain that prevented her from running was eliminated. The subject had correction of a functional leg length discrepancy. This leg length correction occurred during the first three weeks of treatment, which included manual therapy treatment and diathermy treatment. The subject had an incremental decrease in her heel lift from the beginning of treatment until the heel lift was no longer needed at three weeks into the treatment. Her joints were in a soft tissue-contracted position at the start of the study which limited her ROM. Treatment of these soft tissues was successful with our application of pulsed shortwave diathermy and manual therapy techniques. Other researchers have found positive results treating contracted joints with a combination of pulsed shortwave diathermy and manual therapy [8]. The average number of treatments of these individuals was 8 to 13, which is similar to the current study. 

In our study, the subject was treated for 6 weeks to achieve the reported ROM changes. The subject's ROM continued to improve throughout the 6 weeks, but at the end of the six weeks a deficit remained of 15° in hip flexion and hip external rotation ROM between the right and left hips. Ideally this inequality would have been eliminated. Additional treatment would have likely been beneficial, but due to the patient leaving the area, this was not possible. The improved hip ROM was sufficient to allow return to desired activities, such as running and caring for her children; Her ROM improved into a functional range, for example, hip external rotation improved to 30° and according to Perry and Burnfield [9] a total of 15° of rotation is needed in normal gait. The subject was asked to continue with her home exercise program, and it was felt that her joint motion would continue to improve as she maintained her exercises and engaged in functional activities. Many studies have shown an increase in joint motion in a similar time frame in subjects with muscle tightness [10–13]. The subject had more complicated restrictions of hip joint motion. The combined treatment utilized in our study was successful and similar treatment will benefit other patients with complicated soft tissue restrictions, such as individuals who have extensive scar subsequent to debridement surgery for necrotizing fasciitis.

Mortality from necrotizing fasciitis can be reduced by rapid diagnosis, adequate early surgical debridement, and antibiotic treatment [1]. It can occur for a number of reasons including: tooth extraction, dental infections, bone marrow transplant, open and laparoscopic surgery, injection drug abuse, and nonsteroidal anti-inflammatory drug use [2]. Investigators of laryngeal and hypopharyngeal cancer report one patient out of 83 patients suffered an unusual complication of necrotizing fasciitis [3]. This patient required surgical debridement; she was reported to survive the complication with a tracheostomy tube and had returned to work [3]. No other references to individuals with cancer or who underwent chemotherapy treatment that had associated necrotizing fasciitis were found in a search of Medline database and other medical databases. Data about the long term outcomes of surviving the infection are limited. Researchers working with infants in India reported three out of 18 infants (1 death) had extensive scaring of the back and another had long-term functional disability, while the rest of the children had minimal to no scarring and no restriction in the movement of limbs or joints [6].

Individuals with cancer and those who have experienced severe infection can have clinically significant improvement in ROM, in quality of life, and functional outcomes. Our reported condition is rare, and the literature is limited in discussion about the long-term outcomes and treatment of individuals with necrotizing fasciitis. 

One limitation of our treatment was our subjective measurement of her scar softening from our treatment. As we mentioned earlier, the subject had several scars due to her surgeries. Our treatment to soften the scars included PSWD, cross friction massage, and use of massaging the scar tissue with a stainless steel blade to help soften the scar tissues. This seemed to work. Traditionally, tissue firmness or changes in tissue consistency have been performed using subjective palpations and manually applied pressure. Such palpations have been important tools in identifying and evaluating changes in soft tissue abnormalities such as muscle spasticity, atrophy, hypertrophy, localized edema, and scar tissue formation, to name a few. Without objective means of measuring tissue compliance, however, no soft tissue pathological condition can be consistently assessed from clinician to clinician.

## 7. Conclusion

Manual therapy techniques with the use of PSWD and exercise were successful in restoring ROM and improving function in this individual. Many infection and cancer survivors' lives could be improved by following this regimen. In the future, we recommend the scar tissue softening by measured be a more objective manner such as a tissue compliance algometer.

## Figures and Tables

**Table 1:** Joint range of motion (degrees) at baseline, 3 weeks, and 6 weeks of an individual post breast cancer and necrotizing fasciitis while receiving manual therapy, soft tissue mobilization, and diathermy.

	Baseline	3 weeks	6 weeks	Change baseline to 6 weeks
Left Hip External Rotation	20	34	45	25
Right Hip External Rotation	5	20	30	25
Left Hip Flexion	115	120	130	15
Right Hip Flexion	128	132	145	17
Left Hip Extension	25	25	25	0
Right Hip Extension	15	25	25	10
